# Closed-loop systems: recent advancements and lived experiences

**DOI:** 10.1080/17434440.2024.2406901

**Published:** 2024-10-10

**Authors:** Nithya Kadiyala, Roman Hovorka, Charlotte K. Boughton

**Affiliations:** Institute of Metabolic Science-Metabolic Research Laboratories, University of Cambridge, Cambridge, UK

**Keywords:** Automated insulin delivery, fully closed-loop, hybrid closed-loop, type 1 diabetes, type 2 diabetes

## Abstract

**Introduction:**

Hybrid closed loop systems are now commercially available for people with type 1 diabetes and are increasingly being adopted into clinical practice. Real-world data reflect both the glycemic and quality of life benefits reported in trials.

**Areas covered:**

In this review, we summarize the key clinical efficacy and safety evidence for hybrid closed-loop systems, and the lived experience of users with type 1 diabetes across different age groups and during pregnancy. We comment on recent and emerging advancements addressing performance limitations and user experience, as well as the use of closed-loop systems in other types of diabetes.

**Expert opinion:**

Emerging technological developments in closed-loop systems focus on improving performance and increasing automation to further optimize glycemic outcomes and improve quality of life for users. Workforce developments are now urgently required to ensure widespread equitable access to this life-changing technology. Future applications of closed-loop technology are expected to expand into other types of diabetes including type 2 diabetes.

## 1. Introduction

### 1.1. The need for automation

According to data from the type 1 diabetes (T1D) Exchange Clinic Registry, only 26% of people living with T1D met the American Diabetes Association recommended HbA1c goal of <7% in 2021–2022 [1]. With rising global prevalence of T1D, there is a clear need for improved therapies to assist individuals to reach glucose targets to reduce complications and alleviate burden [2]. Despite the increasing use of insulin pumps and glucose sensors, the greatest glycemic and quality of life benefits have been observed with automated insulin delivery systems [3]. This likely relates to the ability of hybrid closed-loop systems (CLS) to manage the high day-to-day variability in insulin requirements of people with T1D [4].

### 1.2. Scope of the review

**Figure 1.**
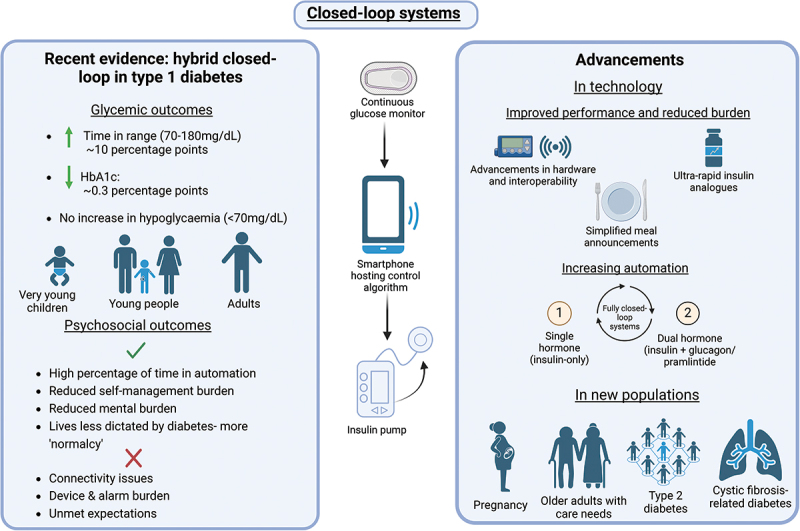
Summary of recent evidence and advancements in closed-loop systems.

In this review, we summarize evidence from randomized controlled trials (RCTs), real-world analyses and qualitative interviews of users of hybrid CLS with T1D across the ages and during pregnancy. We report on recent advancements in closed-loop technology as well as its application in other types of diabetes. Figure 1 provides a summary.

## 2. Closed-loop systems

### 2.1. Components of a closed-loop system

Closed-loop systems use a control algorithm that takes into account real-time interstitial glucose levels from a continuous glucose monitor (CGM) to automatically modulate delivery of insulin via a subcutaneous insulin pump every 5–10 minutes. Reliable communication is required between components using Bluetooth communication. Current CLS algorithms are based on proportional integral derivative (PID), model predictive control (MPC) or fuzzy logic (FL) controllers but often integrate additional components or safety modules resulting in distinct algorithms [5,6].

### 2.2. Commercially available closed-loop systems

**Figure 2.**
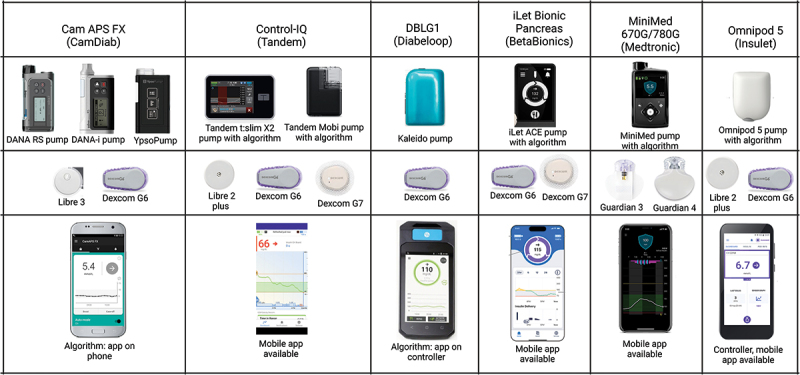
Components of commercially available hybrid closed-loop systems (ordered alphabetically).

All current commercially available CLS are hybrid CLS, which automatically adjust the insulin infusion rate between meals and overnight but require the user to input the carbohydrate content of meals or make a meal announcement for prandial insulin boluses. A hybrid approach with accurate pre-meal boluses is currently required for optimal glycemic outcomes to mitigate the delay in subcutaneous insulin absorption into the bloodstream [7]. Table 1 presents a description of commercially available hybrid CLS and Figure 2 illustrates their components (systems presented in alphabetical order).

**Table 1.** Features of commercially available hybrid closed-loop systems (ordered alphabetically; modified from [72]).

	CamAPS FX (CamDiab, UK)	Control-IQ (Tandem, CA, USA)	DBLG1 (Diabeloop, France)	iLet Bionic Pancreas (BetaBionics, MA, USA)	MiniMed 670 G/780 G (Medtronic, CA, USA)	Omnipod 5 (Insulet, MA, USA)
Algorithm	MPC adaptive algorithm calculates and adjusts insulin sensitivity, carbohydrate bioavailability, and active insulin time.	MPC algorithm uses pre-programmed basal rates and correction factors with automated bolus insulin corrections.	MPC inspired within a physiological framework with an expert system and self-learning algorithms.	MPC adaptive algorithm made up of 3 algorithms (basal insulin, bolus correction & meal aware algorithm).	PID with insulin feedback. 780 G also contains automated bolus insulin corrections.	MPC algorithm calculates adaptive basal insulin rate based on average total daily insulin.
Algorithm setup	TDD, weight	TDD, weight, ICR, CF, basal rate	TDD, weight, typical meal carbohydrate content, basal rate	Weight	TDD, weight, ICR, CF, basal rate	TDD, ICR, CF, basal rate
Adaptive learning	✓ overall, diurnal, meals	X	✓	✓ overall, meal aware algorithm	✓ overall	✓ basal rate for each Pod
Bolusing from phone/handheld device	✓	✓ (with Mobi)	✓	X	X	✓
Personal glucose target	80–200 mg/dL	Default: 112.5–160 mg/dL Sleep mode: 112.5–120 mg/dL Exercise mode: 140–160 mg/dL	100 to 130 mg/dL	Usual: 110 mg/dL Lower: 120 mg/dL Higher: 130 mg/dL	780 G: 100, 110, 120 mg/dL Activity: 150 mg/dL	110 to 150 mg/dL
Activity mode	✓	✓	✓	X	✓	✓
Increased insulin delivery/aggressiveness mode	✓	X	✓	X	X	X
Remote monitoring	✓ All data CamAPS FX Companion feature	✓ CGM only Dexcom Follow app	X	✓ CGM only Dexcom Follow app All data iLet app	780 G: ✓ CareLink Connect app	✓ CGM only Dexcom Follow app Insulin data Omnipod VIEW app
Automated data upload	✓ Glooko	X	✓ YourLoops	✓ Beta Bionics Cloud	780 G: ✓ CareLink	✓ Glooko
Insulin compatibility	Rapid & ultra-rapid	Rapid	Rapid	Rapid & Fiasp	Rapid	Rapid
License	Diabetes requiring insulin age 1+ including pregnancy (FDA and CE mark)	Diabetes requiring insulin age 6+ (FDA and CE mark)	T1D age 18+ (CE mark)	T1D age 6+ (FDA)	T1D age 7+ (FDA and CE mark)	T1D age 2+ (FDA and CE mark)

Abbreviations: CE: Conformité Européenne (European); CF: Correction factor; CGM: Continuous glucose monitoring; FDA: Food and Drug Administration (US); ICR: Insulin:carb ratio; MPC: Model predictive control; PID: Proportional integral derivative; T1D: Type 1 diabetes; TDD: Total daily dose

### 2.3. Open-source closed-loop systems

Alternative ‘Do-it-yourself’ CLS such as OpenAPS, AndroidAPS have been developed by people living with diabetes who wanted to overcome the relative slowness of regulatory approvals of commercial CLS. These open-source systems benefit from the flexibility to adopt advancements from the rapid innovation cycles in this field but lack regulatory quality assurances [8]. Tidepool Loop (Palo Alto, CA, USA) is the first open-source initiative to obtain regulatory clearance (FDA) although it is not yet commercially available [9]. Although open-source systems can allow greater personalization and provide rapid refinements, they require users to have a high level of digital literacy to access and keep up with online communities for support.

## 3. Glycemic outcomes with hybrid closed-loop systems in people with type 1 diabetes

### 3.1. Randomized-controlled trial evidence

#### 3.1.1. Overview

There is a large body of RCT evidence demonstrating the safety and efficacy of hybrid CLS in people with T1D across the ages and during pregnancy. Key RCTs for non-pregnant individuals and during pregnancy are presented in Table 2 and Table 3 respectively, according to the system used (systems reported in alphabetical order). In non-pregnant adults and children, the glycemic outcomes are comparable between systems with improvements in time in range (TIR; 70 to 180 mg/dL) of between 6 and 28 percentage points, and a reduction in HbA1c by between 0.1 and 1.4 percentage points depending on the trial population and control therapy used (e.g. multiple daily insulin injections, sensor augmented pump therapy). Improvements in TIR are achieved without a significant increase in time below range (TBR; <70 mg/dL). Time in automation, i.e. closed-loop usage, is between 71% and 100%.

**Table 2.** Key randomized controlled trials of the commercially available hybrid closed-loop system use in non-pregnant adults and children with type 1 diabetes (systems ordered alphabetically).

Study population	Study design, duration	Comparator	Baseline HbA1c of CL group	Baseline TIR of CL group	Safety outcomes (after randomization)	Change in glycemic outcomes: CL vs comparator (percentage points)	Time in automation	Ref
**CamAPS FX**								
N = 74 Very young children (1–7 yrs)	Parallel, 4 months	SAP	7.3%	62%	SH events: CL: 1, control: 0No DKA events	TIR: ↑ 8.7, TBR: ↑ 0.1 (n.s.),HbA1c: ↓ 0.4	95%	Ware, 2022 [19]
N = 133 Children & adolescents (6–18 yrs) Outcomes reported for the whole cohort (FlorenceM and CamAPS FX configurations)	Parallel, 6 months	Pump	7.9%	50%	SH events: CL: 4, control: 3DKA events: CL: 2, control: 0	TIR: ↑ 6.7 TBR: ↑ 0.5 (n.s.) HbA1c: ↓ 3.5	93%	Ware, 2022 [26]
N = 86 Children, adolescents and adults with sub-optimal glycemic control (≥6 years)	Parallel, 12 weeks	SAP	8.0%	52%	No SH eventsDKA events: CL: 1, control: 0	TIR: ↑ 10.8, TBR: ↓ 0.8, HbA1c: ↓ 0.4	71%	Tauschmann, 2018 [36]
N = 37Older adults (≥60 yrs)	Crossover, 16-week periods	SAP	7.4%	70%	SH events: CL: 0, control: 2No DKA events	TIR: ↑ 8.6, TBR: ↓ 0.1 (n.s.), HbA1c: ↓ 0.2	97%	Boughton, 2022 [42]
**Control-IQ**								
N = 102 Young children (2–6 yrs)	Parallel, 13 weeks	(CSII or MDII) + CGM	7.5%	57%	SH events: CL: 2, control: 1 DKA events: CL: 1, control: 0	TIR: ↑ 12.4, TBR: ↓ 0.2 (n.s.), HbA1c: ↓ 0.4	94%	Wadwa, 2023 [17]
N = 101Children (6–13 yrs)	Parallel, 16 weeks	SAP	7.6%	53%	No SH or DKA events	TIR: ↑ 11.0, TBR: ↓ 0.4 (n.s.), HbA1c: ↓ 0.4 (n.s.)	93%	Breton, 2020 [10]
N = 63 Adolescents and young adults (14–24 yrs)	Parallel, 26 weeks	SAP	8.1%	51%	No SH events DKA events: CL: 1, control: 0	TIR: ↑ 13.0, TBR: ↓ 0.7, HbA1c: ↓ 0.3 (n.s.)	89%	Isganaitis, 2021 [21]
N = 168Adolescents and adults (≥14 yrs)	Parallel, 26 weeks	SAP	7.4%	61%	No SH eventsDKA events: CL: 1, control: 0	TIR: ↑ 11.0, TBR: ↓ 0.9, HbA1c: ↓ 0.3	90%	Brown, 2019 [35]
N = 82 Older adults: ≥65 yrs experiencing hypoglycemia (>1.5% time < 70 mg/dL	Crossover, 12-week periods	PLGS (Basal-IQ), SAP	7.2%	Not reported	Not reported	TIR: vs PLGS: ↑ 7.9, vs SAP: ↑ 8.9; TBR: vs PLGS: ↓ 0.1 (n.s.), vs SAP, ↓ 1.1; HbA1c: vs PLGS: ↓ 0.2, vs SAP: ↓ 0.2	97%	Kudva et al, 2024 [43]
**Diabeloop**								
N = 21 Children (6–12 yrs)	Crossover, 6-week periods	SAP	7.0–7.6%	Not reported	No SH or DKA events	Mean adjusted difference not reported TIR ↑: CL: 66.1% vs control: 58.7%, TBR ↓: CL: 2.6% vs control: 5.2%	100%	Kariyawasam, 2022 [11]
N = 63Adults (≥18 yrs)	Crossover, 12-week periods	SAP	7.6%	Not reported	SH events: CL: 5, control: 3No DKA events	TIR: ↑ 9.2, TBR: ↓ 2.4	84%	Benhamou, 2019 [37]
iLet Bionic Pancreas (insulin only)								
N = 326Children, adolescents, adults (≥6 yrs)	Parallel, 13 weeks	MDII or CSII or SAP or PLGS or HCL	7.9%	51%	SH events: CL: 10, control: 3 No DKA events	TIR: ↑ 11.0, TBR: ↓ 0.1 (n.s.), HbA1c: ↓ 0.5	96%	Russell, 2022 [38]
**MiniMed 670 G HCL**								
N = 18: preschool children (2–6 yrs) N = 20: school children (7–14 yrs)	Crossover, 8 week periods	PLGS	Pre-schoolers: 7.0% School children: 7.7%	Pre-schoolers: 66% School children: 55%	No SH or DKA events	Pre-schoolers TIR: ↑ 5.7, TBR: ↓ 0.3 (n.s.) School children TIR: ↑ 14.0, TBR: ↓ 0.4 (n.s.)	Pre-schoolers 93% School children: 87%	Von dem Berge, 2022 [18]
N = 135Children and adolescents (12–25 yrs)	Parallel, 6 months	(MDII or CSII) with/without CGM	7.8%	53%	No SH or DKA events	TIR: ↑ 6.7; TBR: ↓1.9, HbA1c: ↓ 0.3	Not reported	Abraham, 2021 [24]
N = 252 Children, adolescents, adults (2–80 yrs)	Parallel, 6 months	CSII without CGM	8.3%	53%	SH events: CL: 0, control: 4 No DKA events	TIR: ↑ 12.0, TBR: ↓ 3.6 HbA1c: ↓ 0.6	80%	Garg, 2023 [31]
N = 120Adults (25–70 yrs)	Parallel, 6 months	(MDII or pump) without CGM	7.4%	55%	SH events: CL: 8, control: 7DKA events: CL: 1, control: 2	TIR: ↑ 14.8, TBR: ↓ 2.0, HbA1c: ↓ 0.4	89%	McAuley, 2020 [32]
N = 30Older adults (≥60 yrs)	Crossover, 4-month periods	SAP	7.6%	Not reported	SH events: CL: 3, control: 2 DKA events: CL: 0, control: 1	TIR: ↑ 6.2, TBR: ↓ 0.5, HbA1c: ↓ 0.2 (n.s.)	93%	McAuley, 2022 [41]
**MiniMed 780 G AHCL**								
N = 59Children, adolescents and adults (7–80 yrs)	Crossover, 4-week periods	SAP with PLGM	7.6%	59%	No SH events DKA events: CL: 0, control: 1	TIR: ↑ 12.5, TBR: ↓ 0.4	96%	Collyns, 2021 [33]
N = 113Adolescents and young adults (14–29 yrs)	Crossover, 12-week periods	670 G HCL	7.9%	57%	SH events: CL: 1, control: 0No DKA events	Mean adjusted difference not reported TIR ↑: CL: 67% vs control: 63%, TBR ↔ (n.s.): CL: 2.1% vs control: 2.1%, HbA1c ↓: CL: 7.4% vs control: 7.6%	86%	Bergenstal 2021 [25]
N = 82 Adults with sub-optimal glycemic control (≥18 yrs)	Parallel, 6 months	MDII + CGM	9.1%	36%	No SH or DKA events	TIR: ↑ 27.6, TBR: ↑ 0.1 (p value not reported), HbA1c: ↓ 1 · 4	96%	Choudhury, 2022 [34]

Abbreviations: CGM: Continuous glucose monitoring; CL: Closed-loop; CSII: Continuous subcutaneous insulin infusion; DKA: Diabetic ketoacidosis; HbA1c: Glycated hemoglobin; MDII: Multiple daily insulin injections; N.s.: Not significant; PLGM: Predictive low-glucose monitoring; PLGS: Predictive low-glucose suspend; SAP: Sensor-augmented pump; SH: Severe hypoglycemia; TBR: Time below range (<70 mg/dL); TIR: Time in range (70–180 mg/dL).

**Table 3.** Key randomized controlled trials of commercially available hybrid closed-loop system use in pregnant individuals with type 1 diabetes (systems ordered alphabetically).

Study population	Study design, duration	Comparator	Baseline HbA1c of CL group	Baseline TIRp of CL group	Safety outcomes (after randomization)	Change in glycemic outcomes: CL vs comparator (percentage points)	Time in automation	Ref
**CamAPS FX**								
N = 124 Pregnant women (18–45 yrs, GA< 14 weeks) TIRp, TBRp reported at 16 weeks’ gestation to delivery.	Parallel, until delivery	(CSII or MDII) with CGM	7.6%	48%	SH events: CL: 6, control: 5 DKA events: CL: 1, control: 1	P values not reported for TBRp, HbA1c) TIRp ↑10.5, TBRp ↓ 0.4, HbA1c at 34 to 36 weeks’ gestation: ↓ 0.3	96%	Lee, 2023 [49]
**MiniMed 670 G HCL**								
N = 47 Pregnant women (18–45 yrs, GA< 11 weeks) Results reported at 3^rd^ trimester	Parallel, until 6 weeks postpartum	SAP	6.8%	Not reported	No SH or DKA events.	Mean adjusted difference not reported TIRp: CL: 61.8% vs control: 68.3% (n.s.), TBRp: CL: 2.8% vs control: 5.1% (n.s.), HbA1c: CL: 6.6% vs control: 6.0%	Not reported	Buschur 2023 [47]
**MiniMed 780 G AHCL**								
N = 95 Pregnant women (18–45 yrs, GA < 12 weeks) Results reported at 14 to 36 weeks’ gestation	Parallel, until delivery	(MDII or CSII or PLGS) with CGM	6.5%	61%	SH events: CL: 8, control: 7 DKA event: CL: 1, control: 1	TIRp ↑ 1.9 (n.s.), TBRp ↓ 1.3, HbA1c ↑ 0.1 (n.s.)	95%	Benhalima, 2024 [46]

Abbreviations: CGM: Continuous glucose monitoring; CL: Closed-loop; CSII: Continuous subcutaneous insulin infusion; DKA: Diabetic ketoacidosis; GA: Gestational age; HbA1c: Glycated hemoglobin; MDII: Multiple daily insulin injections; N.s.: Not significant; PLGS: Predictive low-glucose suspend; SAP: Sensor-augmented pump; SH: Severe hypoglycemia; TBRp: Time below range pregnancy (<63 mg/dL); TIRp: Time in range pregnancy (63–140 mg/dL)

There have not been any head-to-head randomized trials comparing the different CLS. Observational analyses comparing systems are limited by the lack of randomization making interpretation of the findings challenging due to underlying differences in the populations using each system [12].

Meta-analysis data including studies eight weeks or longer shows that in children and non-pregnant adults with T1D, the use of CLS increased TIR by ~10 percentage points, decreased TBR by 0.6–1.1 percentage points and HbA1c by ~0.3 percentage points as compared to control therapy with no increase in the risk of diabetic ketoacidosis (DKA) or severe hypoglycemia [13,14]. The benefit of CLS over non-automated therapies on glucose outcomes is predominantly overnight compared to daytime due to the absence of factors such as physical activity and meals [7,15].

#### 3.1.2. Very young children

Very young children with T1D present unique diabetes management challenges for caregivers due to unpredictable food intake and physical activity, difficulties communicating symptoms of hypoglycemia, as well as high variation in day-to-day insulin requirements [4,14,16]. In this vulnerable population, RCTs of hybrid CLS using the CamAPS FX, Control-IQ and MiniMed 670 G systems compared to control showed an increase in TIR of 8–12 percentage points, no increase in TBR, and a reduction in HbA1c of ~0.4 percentage points with no safety concerns [17–19]. CLS usage was 93–95% suggesting high acceptability in this population. Generalizability of the study findings are limited by the lack of diversity in the trial populations and low baseline HbA1c (7.0–7.5%). At present, only CamAPS FX and Omnipod 5 are approved for use in children under 7 years old (Table 1).

#### 3.1.3. Adolescents and young adults

Adolescence can be a challenging developmental stage and people with T1D in this age group often have suboptimal glucose outcomes and high levels of diabetes distress [20]. Registry data from the United States and northern Europe shows that HbA1c increases at adolescence and remains high into early adulthood [21,22]. This may be due to difficulties engaging with diabetes self-management mental health challenges, and physiological endocrine changes associated with puberty [23].

Evidence from RCTs, including subgroup analyses of broader trials, using the CamAPS FX, Control-IQ, MiniMed 670 G and MiniMed 780 G hybrid CLS demonstrate an increase in TIR of 7 to 13 percentage points, no increase in TBR and a reduction in HbA1c of 0.3 percentage points compared to control therapy [21,24–26]. Supporting acceptability of hybrid CLS in this population is usage of between 86% and 89%. The importance of ease of use of the technology has been highlighted in two trials where improved CLS operation was associated with better glucose outcomes [25,26]. Trials involving adolescents and young adults have also shown that lower baseline TIR and/or higher baseline HbA1c are associated with the greatest improvements in glycemic outcomes with hybrid CLS [7,27].

Recent RCTs involving children and adolescents recently diagnosed with T1D have shown that hybrid CLS soon after diagnoses of T1D is safe and effective in improving glucose outcomes compared to standard care [28,29]. Although hybrid CLS use for up to four years did not prevent the decline in C-peptide levels that occurs after diagnosis, TIR increased by 12–16 percentage points, TBR did not significantly increase and HbA1c reduced by 0.7–0.9 percentage points. CLS usage was 93–97%. Furthermore, secondary analysis of data from the CLOuD study suggests that hybrid CLS use from diagnosis may mitigate the negative glycemic effects associated with presentation in DKA at T1D diagnosis [30].

#### 3.1.4. Adults

RCTs in adults showed an increase in TIR of 9 to 28 percentage points, no increase in TBR and a reduction in HbA1c of 0.3 to 1.4 percentage points compared to control therapy [31–38]. Time in automation was between 71% and 96%.

#### 3.1.5. Older adults

Older adults with T1D are more likely to be affected by comorbidities, polypharmacy, cognitive impairment, functional dependence and frailty compared to younger counterparts. They are at greater risk of severe hypoglycemia and its consequences including acute changes in cognition, seizures, falls and fractures [39,40]. RCTs in adults aged over 60 years, using the CamAPS FX, Control-IQ and MiniMed 670 G systems, showed that hybrid CLS compared to sensor augmented pump therapy increased TIR by 6–9 percentage points, decreased TBR by 0.1 to 1 percentage points and decreased HbA1c by 0.2 percentage points [41–43]. CLS usage was 93–97%. An important limitation of these studies is the lack of frail participants with significant cognitive impairment. Future studies should determine whether this technology might be beneficial in more vulnerable, dependent older adults where the responsibility for diabetes management is with caregivers.

#### 3.1.6. Pregnancy

Type 1 diabetes management during pregnancy is challenging due to metabolic changes and marked gestational variation in insulin sensitivity. More stringent glucose targets for TIR during pregnancy (TIRp; 63–140 mg/dL) are recommended to reduce the risk of diabetes-related complications for mother and fetus such as pre-eclampsia, preterm delivery, neonatal intensive care admissions and congenital abnormalities [44]. Every increase in time in target range of five percentage points is associated with improved pregnancy outcomes, but tight glycemic control may increase the risk of maternal hypoglycemia [45,46].

Most commercially available hybrid CLS have been designed for non-pregnant populations and the algorithm glucose target cannot be lowered <100 mg/dL which may limit the extent to which tighter pregnancy glucose targets can be met (Table 3) [47,48]. The closed-loop algorithms also need to adapt to the increased insulin need later in pregnancy [48]. CamAPS FX CLS has been designed to accommodate the demands of pregnancy with a wide range of glucose targets as low as 80 mg/dL, and highly adaptive algorithm. It is the only CLS authorized for use in this population. The AiDAPT RCT showed a 10.5 percentage point increase in TIRp with CamAPS FX compared to standard insulin therapy with CGM with no increased TBRp from 16 weeks of pregnancy to delivery [49]. HbA1c at 34–36 weeks’ gestation was 0.3 percentage points lower in the CLS group compared to control group. CLS usage was 96%. The CRISTAL study using the MiniMed 780 G system showed conflicting results, showing no improvement in TIRp or HbA1c compared with standard insulin therapy at 33–36 weeks’ gestation. The lowest target glucose setting in this system is 100 mg/dL.

It appears that apart from the CamAPS FX system, other commercially available hybrid CLS algorithms cannot be assumed to safely and effectively manage pregnancy requirements and may require further refinement [48]. The impact of hybrid CLS use in the postpartum period and during breastfeeding has not yet been reported and is an area of clinical interest.

### 3.2. Real-world data

Evidence from RCTs has been essential in securing regulatory approval for hybrid CLS. However, the findings from these trials are limited by the inclusion of relatively motivated users who additionally receive the support and attention of specialist study teams. Furthermore, the socioeconomic and ethnic diversity of trial populations often do not reflect that of the general population with T1D. Despite this, the efficacy and acceptability of CLS have encouragingly been replicated in real-world settings.

A systematic review of retrospective real-world studies in over 170,000 non-pregnant people with T1D showed a 10 percentage point increase in TIR, no change or reduction in TBR and 0.1–0.9 percentage point decrease in HbA1c compared to before hybrid CLS [50]. Issues with usability of the first-generation MiniMed 670 G hybrid CLS are reflected in a time in automation mode of between 63% and 90%, while hybrid CLS usage with CamAPS FX, Control-IQ, DBLG1, the second generation MiniMed 780 G and open-source systems was 90–99%. Real world data also identified factors associated with a higher TIR with hybrid CLS including more frequent user-initiated boluses, lower glucose target settings, and increased time spent in automation mode [51–53].

## 4. Psychosocial outcomes of hybrid closed-loop system use in people with type 1 diabetes

### 4.1. Questionnaires

Psychosocial evaluations of hybrid CLS using questionnaires have yielded inconsistent results, perhaps due to differences in study design, and length of the study follow-up period [54,55]. Meta-analyses of RCTs using CLS for longer than eight weeks showed some or no improvement in patient distress and no difference in treatment satisfaction or fear of hypoglycemia [13,14]. The lack of positive findings may be attributed to the lack of refinement of the questionnaires, the burden of starting a new technology or adhering to trial requirements, which can disrupt a person’s routine.

### 4.2. Lived experiences

Several qualitative studies involving focus groups and semi-structured interviews have assessed the impact of hybrid CLS use in people with T1D. These studies provide a richer, more detailed understanding of how the technology impacts quality of life [56]. Perceived benefits of CLS use across different age groups and in pregnant individuals with T1D relate to reduced self-management and mental burden of diabetes allowing users to lead lives less dictated by diabetes [57–61]. Reported limitations of hybrid CLS include issues with device connectivity, alarms and device burden, and unmet expectations.

#### 4.2.1. Perceived benefits

The perceived glycemic improvements with hybrid CLS led to less anxiety and concern over long-term complications, and pregnant users specified that they were less worried about risks to the fetus [56,59,61,62]. The ability of hybrid CLS to maintain glucose levels in target range allowed users to reduce the time spent managing diabetes and relieved some of the associated mental burden [62]. Hybrid CLS users reported that more stable glucose levels also resulted in fewer disruptive alarms overnight and improved the amount and quality of sleep, although this has not been consistently demonstrated in studies specifically evaluating the effect of hybrid CLS on sleep [55,57,58,60,63].

Users described how the ability of hybrid closed-loop to adapt to changes afforded them more flexibility in their lifestyle, particularly regarding food choices and eating out [20,59,62,64]. Parents of very young children noted benefits of the CLS adjusting to their child’s unpredictable meal intake or accounting for missed boluses or inaccurately counted carbohydrates [58].

Caregivers of children using hybrid closed-loop reported that its use enabled their child to take part in more ‘normal’ activities. They felt more comfortable spending time apart from children allowing them to have playdates, sleepovers and go out unsupervised [57,58,63,65]. They also entrusted others, e.g. teachers and friends’ parents to oversee diabetes management. The reduction in caregiving responsibilities meant they had more time and energy to dedicate to their own and everyday family life resulting in more time spent with their partner and other children and some people even returning to employment [58]. Older adults stated that the CLS minimized the risk of severe hypoglycemia so relatives felt more comfortable with them living alone or looking after grandchildren [62]. Hybrid closed-loop facilitated more normalcy in pregnancy with some users being able to work longer than in previous pregnancies and having more time to devote to childcare of older children and other family activities [61].

The availability of CLS data to healthcare providers was generally perceived to be positive, enabling convenient remote consultations and detailed feedback to optimize self-management [61,64,66,67]. However, some people felt a loss of privacy at their data being viewed and were concerned about facing judgment from healthcare providers [61].

#### 4.2.2. Perceived limitations

Users reported frustrations related to technical issues with hardware components and device communication, including poor sensor connectivity, sensor inaccuracy and pump infusion set failures [20,59,63,66,68,69]. Some users highlighted disruption to social life and sleep due to excessive alarms, some of which are triggered by sensor inaccuracy. Highlighting the importance of usability, real-world data from the first-generation Medtronic 670 G hybrid closed-loop system showed discontinuation rates of 33% 12 months after starting the technology in adults ([70]) and 30% at 6 months in children [71]. Primary reasons for discontinuation include technological difficulties e.g. sensor error alerts, too much work with calibrations and fingerstick glucose checks and excess alarms. The second-generation Medtronic system (780 G) improved many of these factors and has much greater usability.

Another limitation described was the burden of wearing and carrying multiple devices [66]. The opportunity to bolus from a smartphone as opposed to bolusing from the pump was perceived to be easier and more discreet but may be associated with additional device burden if a compatible smartphone is not the user’s primary smartphone [65–67]. The visibility of devices remains a barrier, particularly amongst young people [69].

Some users experienced unmet expectations when they anticipated the algorithm would ‘learn’ faster and they found the system did not adjust quickly enough [59]. Some individuals reported hybrid CLS to be more cautious than they expected when responding to hyperglycemia, resulting in frustration and a perceived need for manual insulin correction boluses [20,57,59,62]. It is clear that hybrid CLS are not simply ‘plug and play’ systems but require ongoing user engagement for optimal outcomes and healthcare providers should discuss realistic expectations with new users prior to initiation [7].

### 4.3. Future directions

Reports of user experiences are limited by the relative lack of ethnic and socioeconomic diversity of the research study populations [72]. In depth reports of usability from real-world settings are also lacking and are important for consideration of widening access to CLS and improving outcomes in the wider, more diverse population with T1D.

As closed-loop technology is rapidly evolving and each CLS has unique features, the use of consistent person-reported outcome measures may help to understand the distinct advantages and limitations of each CLS for particular populations, and to inform future innovations in the field [54,55].

## 5. Advancements in CLS

### 5.1. Advancements in hardware and interoperability

Most glucose sensors now do not require fingerstick glucose calibration and are smaller with longer duration of wear-time than previous generations of sensors [12,73]. The integration of patch pumps (e.g. Omnipod) and smaller pumps with shorter tubing (e.g. Tandem Mobi) offer users more options to choose a hybrid CLS to suit their needs. Infusion sets lasting up to seven days have recently received regulatory approval having shown safety without adversely affecting glycemic control in adults with T1D, and improved user satisfaction [74]. Commercial hybrid CLS are increasingly providing users with the option to view glucose levels and deliver insulin boluses remotely through a smartphone or smartwatch.

Future developments should include more interoperability between devices which would allow users a wider choice of CLS components to suit their needs. Single integrated devices which combine an insulin pump with a glucose sensor in one platform are being pursued although currently these are limited by concerns about catheter leakage and sensor accuracy but have the potential to reduce the on-body burden if successful [75].

### 5.2. Advancements to improve post-prandial glucose excursions

#### 5.2.1. Simplified meal announcements

Accurate carbohydrate counting can be challenging, burdensome and error prone. It may also encourage people to limit their dietary choices or choose prepackaged food over potentially healthier wholefoods due to ease of nutrition labels [76]. Simplified meal announcements use qualitative meal-size estimation requiring users to select a preset meal size such as (‘small,’ ‘medium’ or ‘large’). Although simplified meal announcements may compromise glucose outcomes compared to precise carbohydrate counting, the average TIR was above 70% in small feasibility RCTs including adults and adolescents with T1D using CLS which employ this approach in outpatient free-living settings [76,77]. The insulin-only bionic pancreas (BP) system (iLet, Beta Bionics) uses qualitative meal announcements of ‘Less,’ ‘Usual for me’ and ‘More’ and is uniquely initialized with only body weight, without requiring pre-programming or adjustment of insulin settings, such as insulin to carbohydrate ratios, by the user or healthcare provider [38]. Despite the simplified meal announcements, in the pivotal trial involving over 200 participants aged 6–79 years, glucose outcomes in the CLS group were superior to the control group; TIR was 65% vs 54% respectively [38].

Another novel approach under development is a meal detection app to reduce post-prandial hyperglycemia due to missed or late boluses [3,78]. The smartphone-paired smartwatch with the Klue application (Klue, Inc., BC, Canada) detects eating gestures using built-in motion sensors and converts these gestures into bite-sized carbohydrate amounts (1–10 g). Studies undertaken in supervised settings demonstrate the feasibility of this approach but longer free-living studies are required to determine efficacy and safety [79]. Future advancements may integrate other alternatives to manual carbohydrate counting, e.g. smartphone apps that can process images of meals to derive carbohydrate content [80].

#### 5.2.2. Adjuncts

Adjunctive medications such as glucagon-like peptide 1 (GLP-1) receptor agonists and sodium glucose transporter 2 (SGLT-2) inhibitors administered alongside CLS to target post-prandial glucose control and potentially allow a fully closed-loop approach show feasibility although there are concerns around an increased risk of DKA in adults with T1D [72,81,82].

#### 5.2.3. Ultra-rapid insulin analogues

Several RCTs investigating the use of ultra-rapid insulins in CLS have been undertaken with conflicting results [78]. Studies show either comparable or modestly improved efficacy with ultra-rapid insulins compared to rapid acting insulin analogues in terms of TIR and time in hypoglycemia [78,83,84]. Discrepancies in outcomes are likely due to differences in closed-loop algorithms, study design and/or study populations.

Alternative routes of insulin delivery, including intraperitoneal and inhaled insulin, are being investigated and the more rapid absorption compared to the subcutaneous route may counteract postprandial hyperglycemia and minimize hypoglycemia [85,86].

### 5.3. Increasing automation

Fully CLS are designed to not require any mealtime announcement by users, thus decreasing the burden of diabetes management. A recent study showed that CamAPS HX fully CLS (CamDiab, UK) with ultra-rapid insulin lispro (Lyumjev) was safe and improved glycemic control compared to pump and sensor therapy in adults with T1D and suboptimal glycemic control (HbA1c >8%) [87]. It remains to be seen whether fully closed-loop algorithms can be further optimized to allow for single hormone (insulin only) fully CLS.

Dual-hormone CLS delivering insulin and glucagon or insulin and the amylin analogue, pramlintide, aim to more closely imitate pancreatic glucose control physiology and may improve performance with regards to managing postprandial glucose excursions and exercise-induced hypoglycemia [88].

Systems that deliver glucagon in addition to insulin can be more effective during, and in preventing hypoglycemia episodes than insulin delivery alone. They also allow for more aggressive insulin delivery potentially reducing hyperglycemia and improving glucose outcomes. The insulin and glucagon dual-hormone fully CLS developed by Inreda Diabetic B.V. (Goor, the Netherlands) requires two pumps with separate infusion sets and two sensors [89]. During a 1 year prospective, single-arm trial in 79 adults with T1D, TIR was 80%, TBR was 1.4% and HbA1c was 6.9%. Of note, the use of glucagon was associated with relatively frequent symptoms of nausea, infusion site reactions and set occlusions. The discontinuation rate (10/79) indicates the burden of using the system. Alternative formulations of glucagon might yield a more practical and acceptable approach. A short crossover study in 10 adults with T1D compared the dual-hormone iLet with insulin and dasiglucagon, a chemically stable glucagon analogue (Zealand Pharma), with the insulin-only iLet over a seven-day period in the home setting [90]. TIR was 79% and 72% respectively and there were no episodes of severe hypoglycemia or diabetic ketoacidosis. Only one prefilled dasiglucagon cartridge was required for the entire 7-day period and no infusion site reactions or occlusions occurred [90].

Amylin is normally co-secreted with insulin by pancreatic beta-cells, and in T1D there is an amylin deficiency because of beta-cell destruction [91]. Amylin slows gastric emptying and suppresses glucagon postprandially, reducing glucose excursions. Short RCTs undertaken in supervised settings have shown that insulin-and-pramlintide (amylin analogue) dual-hormone CLS achieved a time in range >70% [91–93]. One limitation in the use of pramlintide is gastrointestinal symptoms such as nausea, vomiting and bloating although these are typically transient and mild to moderate [91,94]. Coformulations of insulin and pramlintide and dual-chamber pumps are under development, which would remove the need for a second pump [95].

## 6. CLS in other populations with diabetes

### 6.1. Type 2 diabetes

The global burden of type 2 diabetes (T2D) is increasing and due to the earlier age of onset, the prevalence of micro- and macrovascular complications is also rising. Despite the introduction of new therapies such as the GLP-1 receptor agonists and SGLT2 inhibitors, many people with T2D do not meet the recommended glucose targets and still require insulin to manage their diabetes [96].

Off-label use of hybrid CLS is increasing in people with T2D, particularly in the US [97]. These CLS require the user to calculate insulin doses for meals and need ongoing healthcare provider input for optimization, therefore may not be suitable for the wider population with T2D, and there may be insufficient healthcare provider resources to meet the demand. A fully CLS (CamAPS HX) has been developed specifically for people with T2D either for use in the acute hospital setting or in the home setting [98–100]. The fully closed-loop approach does not require any meal announcements and removes the need for ongoing healthcare professional input after initial device training. Small RCTs in adults with T2D have shown dramatic improvement in glucose outcomes compared to standard insulin therapy without an increased risk of hypoglycemia [99–101]. There are limited reports of the psychosocial impact of CLS in people living with T2D at present. Given the high prevalence of T2D, understanding who will benefit most from this approach will be important to manage the workload burden for healthcare professionals and the potential economic implications for healthcare systems.

### 6.2. Cystic fibrosis-related diabetes (CFRD)

Diabetes is the commonest complication of cystic fibrosis and the prevalence is rising due to the widespread introduction of modulator therapies and improved life-expectancy. Suboptimal glucose control in people with CFRD is associated with a decline in lung function and body weight, and increased mortality [102]. Insulin management of CFRD can be difficult due to highly variable daily insulin requirements secondary to malabsorption associated with exocrine pancreatic dysfunction, frequent pulmonary exacerbations requiring corticosteroids and supplementary nutrition requirements [103]. Highly adaptive CLS which can be used with ultra-rapid insulins are an attractive option to optimize glycemic outcomes and reduce burden in this population. Small studies have shown feasibility of CLS in people with CFRD [104,105]. A crossover RCT involving 20 adults with CFRD showed glycemic outcomes were improved using the insulin-only iLet Bionic Pancreas compared to usual care (TIR: 75% vs 62%), with no increase in hypoglycemia [102]. There are limited reports of the psychosocial impact of CLS in people living with CFRD at present [72].

### 6.3. Future directions

Larger studies over a longer duration are needed to understand the impact of CLS on long-term glycemic outcomes and quality of life. As CLS have primarily been designed for people with T1D, this would inform potential algorithm adjustments required to accommodate the specific needs of these individuals [106]. There are no in-depth reports of the psychosocial impact or cost-efficacy of CLS in T2D and people with CFRD and this warrants further research [72]. The training needs of healthcare professionals supporting these individuals also requires consideration.

## 7. Implementation and access

With the increasing number of commercially available hybrid CLS, people with T1D should be able to choose the system that best meets their needs. However, this poses increasing challenges for healthcare providers in supporting users, who need to be familiar with each of the different systems. Adequate time and training is required to educate a specialist workforce to implement CLS effectively. The is exemplified by the UK National Institute for Health and Care Excellence (NICE) approving the use of CLS in all children, young people, pregnant women, and other adults, the latter meeting specific inclusion criteria, with a phased rollout planned over five years [107].

Healthcare professionals often act as gatekeepers to diabetes technology access and widespread use depends on open-mindedness and availability of healthcare teams to support users, particularly those from underserved groups [108,109]. Existing disparities in access to diabetes technology are well documented in those from lower socioeconomic and ethnic minority backgrounds [1]. Further work is required to identify contributors to this and implement effective interventions to reduce the gap. Recruitment of more diverse study participants in future research studies would also yield more generalizable outcomes.

Much of the research on CLS use has been undertaken in high-income countries, and uptake is highest in these settings **[**110**]**. Barriers to wider adoption of CLS globally include lack of government reimbursement, high cost and inadequate infrastructure to implement technology use in areas with poorer healthcare provision. Long-term studies providing cost effectiveness data may support wider government reimbursement and ensure more widespread access.

## 8. Conclusions

Hybrid CLS are increasingly being adopted into routine clinical care for people with T1D. RCT and real-world data provide compelling evidence of the glycemic and quality of life benefits of hybrid closed-loop in children and adults with T1D. Further research involving specific populations such as pregnant women with diabetes, older adults with T1D who have care needs, adults with T2D, and people with CFRD is required to fully understand the role of CLS in these groups. Ongoing challenges to existing CLS include reducing device and alarm burden, improving interoperability between devices and optimizing management of post-prandial glucose excursions. CLS advancements under development include reducing burden with simplified meal announcement, improving performance with ultra-rapid insulin analogues, and increasing automation toward a fully closed-loop approach with adjunctive therapies and dual hormone systems. Future research studies must include more representation from underrepresented groups in the study population to ensure effective implementation of this technology and reduce health-related disparities. This might be achieved by increasing the diversity of the research team, explicitly addressing healthcare professional bias and prioritizing individuals from underserved backgrounds.

## 9. Expert opinion

Hybrid closed-loop systems are now being widely adopted as part of routine clinical care for people with type 1 diabetes, supported by an abundance of clinical trial evidence of the glycemic and quality of life benefits. The emerging real-world data is consistent with the trial findings, across a wider population following implementation in routine clinical practice. People with type 1 diabetes now have a choice of closed-loop systems to suit their individual preferences and needs, however maintaining professional competency in so many different closed-loop system to be able to educate and support users can be challenging for already stretched healthcare providers. Improved interoperability between devices allows for even more flexibility and customization for users and likely improves the user experience.

Emerging closed-loop systems are targeting reduced burden and improved performance around mealtimes with use of simplified meal announcements and ultra-rapid insulins, respectively. Integration of additional signals such as meal detection inputs, continuous ketone monitoring or heart rate monitoring may improve performance but needs to be accurate and reliable enough to prevent additional burden for the user. Advancement toward a fully closed-loop approach, without compromising glucose outcomes is key to truly reduce the burden of diabetes self-management and improve quality of life. Current approaches under development include use of adjunctive medications alongside closed-loop systems and dual-hormone systems with insulin and glucagon or insulin and pramlintide but both approaches are limited at present by additional device complexity and/or potential side-effects.

The relatively rapid translation of closed-loop systems into clinical practice has revealed important disparities in access to technologies which is contributing to inequalities in health outcomes. Individuals from underserved populations, such as those from lower socioeconomic groups or ethnic minority backgrounds, have not been adequately represented in clinical trials of closed-loop systems to date and healthcare providers often act as gatekeepers, with (un)conscious biases negatively affecting those from such backgrounds [111]. Future trials need to ensure adequate representation of people from underserved backgrounds to better understand any specific training and support needs to ensure successful implementation. Other barriers impacting on equitable access include upskilling the current healthcare professional workforce to be able to effectively implement closed-loop systems, addressing clinical inertia and securing widespread reimbursement. Current reimbursement policies are highly variable by country and existing policies need to be updated to broaden access across all age-groups with type 1 diabetes.

Further research is required to fully understand the role of hybrid closed-loop in pregnant women with type 1 diabetes including during the post-partum period, and in older adults who have care needs. Expanding the application of closed-loop systems to other populations with diabetes is increasingly becoming the focus of future research in this field. With the anticipated rise in prevalence of type 2 diabetes and earlier age of onset, it is likely that closed-loop systems will have an important role in management of those requiring insulin. More automated (fully closed-loop) systems which do not require much user education or healthcare provider input will be essential to prevent overwhelming the current workforce. These closed loop systems would likely need to be initiated in the primary care setting so future studies should explore implementation of this technology within these settings [112].

Closed-loop systems have significant and sustained clinical benefits for people with type 1 diabetes; long term data will be crucial to determine how this technology can impact on both acute and chronic (micro and macrovascular) complications of diabetes.

## Article highlights

- Hybrid closed-loop systems are now commercially available and increasingly being used in clinical care for children, adults and pregnant people with type 1 diabetes.
- These systems have been shown to improve glycemic outcomes for people with type 1 diabetes both in clinical trials and real-world settings.
- Patient-reported outcomes measured by questionnaires have been inconsistent but reports of the lived experiences of people with type 1 diabetes indicate improved quality of life with some limitations around device connectivity and unmet expectations.
- Emerging advancements under development aim for improved performance with ultra-rapid insulin analogues, adjuncts or bihormonal systems, reduced burden with simplified meal announcements and increased automation with a fully closed-loop approach.
- Further research expanding the application of closed-loop systems to include other types of diabetes, e.g. type 2 diabetes and cystic fibrosis-related diabetes is needed to understand the benefits of this technology in these user groups.
- A specialist workforce, adequate infrastructure and ongoing research including inclusion of underrepresented groups is required to successfully and equitably implement closed-loop systems in routine clinical practice.
